# Physiological and Molecular Timing of the Glucose to Acetate Transition in *Escherichia coli*

**DOI:** 10.3390/metabo3030820

**Published:** 2013-09-20

**Authors:** Brice Enjalbert, Fabien Letisse, Jean-Charles Portais

**Affiliations:** 1 Université de Toulouse; INSA, UPS, INP, 135 Avenue de Rangueil, Toulouse F-31077, France; E-Mail: brice.enjalbert@insa-toulouse.fr; 2 INRA, UMR792 Ingénierie des Systèmes Biologiques et des Procédés, LISBP, Toulouse F-31400, France; E-Mail: Fabien.letisse@insa-toulouse.fr; 3 CNRS, UMR5504, Toulouse F-31400, France

**Keywords:** *E. coli*, carbon metabolism, transition, metabolome, transcriptome

## Abstract

The glucose-acetate transition in *Escherichia coli* is a classical model of metabolic adaptation. Here, we describe the dynamics of the molecular processes involved in this metabolic transition, with a particular focus on glucose exhaustion. Although changes in the metabolome were observed before glucose exhaustion, our results point to a massive reshuffling at both the transcriptome and metabolome levels in the very first min following glucose exhaustion. A new transcriptional pattern, involving a change in genome expression in one-sixth of the *E. coli* genome, was established within 10 min and remained stable until the acetate was completely consumed. Changes in the metabolome took longer and stabilized 40 min after glucose exhaustion. Integration of multi-omics data revealed different modifications and timescales between the transcriptome and metabolome, but both point to a rapid adaptation of less than an hour. This work provides detailed information on the order, timing and extent of the molecular and physiological events that occur during the glucose-acetate transition and that are of particular interest for the development of dynamic models of metabolism.

## 1. Introduction

The Enterobacterium, *Escherichia coli*, is subjected to constant environmental variation in the intestine, especially in nutrient availability [1]. The capability of the bacterium to efficiently alternate nutrients, which requires the reorganization of cellular metabolism, is a fundamental advantage in the competition with other colonic microorganisms [2,3,4]. The metabolic flexibility of *E. coli* is also extensively exploited for a broad range of biotechnological applications in which metabolism can be modified to enable the production of valuable compounds. Hence, a comprehensive understanding of the mechanisms that ensure efficient adaptation of metabolism to nutrient changes is of great interest for both basic and applied research [5]. The glucose-acetate switch is a classical model of metabolic transition in *E. coli*, partly because of its physiological importance [6] and partly because the production of acetate as a fermentation by-product can be a major obstacle in the development of biotechnological processes. During the growth of *E. coli* on glucose, a significant proportion of the sugar is converted into acetate. This process results from an overflow metabolism that is usually explained by saturation of the tricarboxylic acid cycle and the need to regenerate NAD^+^ [6]. When glucose becomes scarce, *E. coli* cells use the previously produced acetate. This requires a profound reorganization of their central carbon metabolism, mainly the shutdown of glycolysis, the activation of acetate utilization pathways and the activation of gluconeogenesis. Because it involves network-wide metabolic adaptation, the glucose-acetate transition is also the current paradigm in systems biology for the development of explanatory and predictive models of metabolism and its adaptation [7,8,9].

The molecular mechanisms underlying the adaptation of metabolism to glucose and acetate have been extensively studied; for a review, see [6,10]. In contrast, the dynamics of the metabolic transition is poorly understood with respect to the sequence and timing of the molecular events involved in the reorganization of metabolism. *E. coli* cells pre-grown on glucose and transferred into fresh medium containing acetate as the sole carbon source needed more than three hours for a novel gene-expression pattern to appear that allowed them to grow on the new substrate [11,12,13]. In a more recent study of the response of glucose-limited chemostat cultures to glucose pulses, Sunya *et al.* [14] suggested that a metabolic response could occur within two to three min. These investigations showed that the timing of the metabolic transition is still elusive. Neither has there been a detailed investigation of the timing of molecular and metabolic processes during the adaptation phase, despite the fact that this transition is a valuable biological framework for the development of dynamic metabolic models in systems biology [8,15]. Here, we report a detailed investigation of the timing of transcriptome and metabolome changes during the glucose-acetate transition in a standard culture of *E. coli* on glucose. The results showed the overall rapidity of the transition, which was completed within 40 min after glucose exhaustion, and most of the molecular components adjusted in the first 10 min. Finally, incorporating multi-omics data revealed different changes and timescales between the transcriptome and the metabolome.

## 2. Results and Discussion

### 2.1. Results

#### 2.1.1. Macrophenotype Parameters of the Glucose/Acetate Transition in a Model Condition

**Figure 1:**
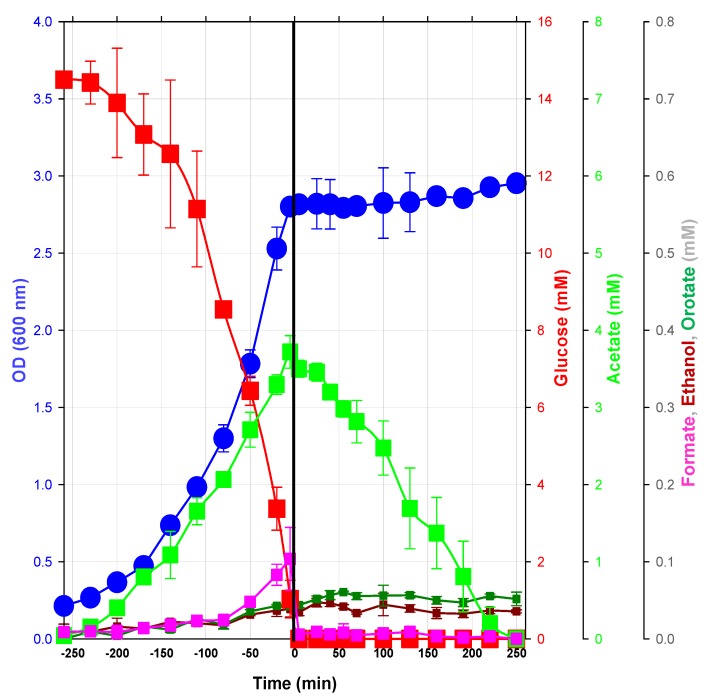
Physiological characterization of the glucose/acetate transition in *E. coli*. Three independent cultures of *E. coli* were grown in a bioreactor as described in the Experimental section. Time 0 was defined as the time (±30s) when the pO_2_ suddenly increased, indicating complete consumption of glucose. Culture samples were collected every 10 to 30 min to measure growth (OD: Optical Density at 600 nm) and extracellular metabolite concentrations.

Detailed physiological characterization of the glucose/acetate transition was obtained from *Escherichia coli* K12 MG1655 cells grown in M9 mineral medium [16] containing 15 mM glucose (2.7 g L^−1^) as the sole carbon source. Growth experiments were performed aerobically in bioreactors at 37 °C, pH 7, under sufficient oxygenation (pO_2_ > 20%). As expected, cells consumed glucose for growth, which followed an exponential regime, as long as glucose was available in the medium (Figure 1). Acetate was produced during growth on glucose, with the maximum concentration being observed when glucose was completely consumed. Besides acetate, other metabolic end products, such as formate, ethanol and orotate, were detected by nuclear magnetic resonance (NMR) in the culture medium collected during the exponential growth phase. The maximum concentrations of formate, ethanol and orotate were 20-times lower than that of acetate (Figure 1). Orotate is an intermediate in the *de novo* biosynthesis pathway of UMP, and its accumulation is explained by its limited ability to synthesize purines in some *E. coli* strains, including in the strain, K12 MG1655 [17]. The detection of this compound illustrates the advantage of NMR in detecting metabolic end products without prior consideration. Because all products were correctly identified and quantified by NMR, a nearly complete carbon balance was obtained at the time of glucose exhaustion (95.3%, encompassing: biomass, 54.8%; CO_2_, 31.0%; acetate, 8.6%; orotate, 0.6%; ethanol, 0.2%; and formate, 0.04%). Past this point, acetate was consumed for three hours before exhaustion. The small amount of formate detected at the end of the exponential growth phase was readily consumed, whereas neither orotate nor ethanol was consumed over the entire study period. For the sake of simplicity, the period of culture will be expressed relative to the time of glucose exhaustion (GE) in the following (Figure 1). *Before GE* refers to the period during which cells grow exponentially using glucose as the carbon source. *After GE* refers to what occurs subsequently.

#### 2.1.2. Changes in Metabolite Profiles

**Figure 2:**
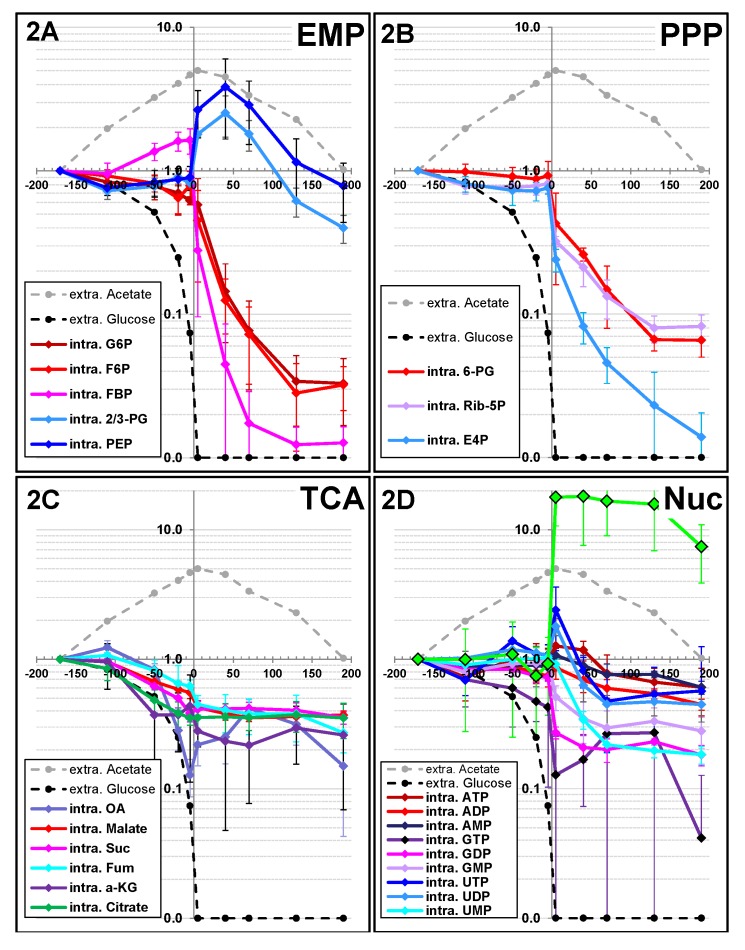
Changes in metabolite pool sizes during the glucose/acetate transition. Intracellular contents of central carbon metabolites were determined as described in the Experimental section. For the sake of clarity, the metabolites were divided into four groups according to the metabolic pathways to which they belong: the Embden-Meyerhof-Parnas pathway (glycolysis, **2A**), the pentose phosphate pathway (metabolites, **2B**), the tricarboxylic acid pathway (metabolites, **2C**) and nucleotides and nucleotide-derived molecules (**2D**). Ratios were obtained by normalizing the concentrations to a sample collected three hours before glucose exhaustion, *i.e.*, during early exponential growth.

The metabolic events that occur during the diauxic transition were investigated by measuring changes in the time course of metabolite pools. Metabolites belonging to central carbon pathways (mainly glycolysis, the PP pathway, the ED pathway and the TCA cycle) and to energy metabolism (*i.e.*, nucleotides) were analyzed by ion-exchange chromatography coupled with tandem MS (IC-MS/MS; [18,19]). Reliable quantification of metabolite pools was obtained by isotopomer dilution mass spectrometry [20]. To obtain detailed information about the changes in metabolite pools during the metabolic transition, metabolic samples were collected throughout the culture period until the acetate was exhausted. The pool sizes of central carbon metabolites measured during the exponential growth phase were consistent with results obtained with *E. coli* cells grown in similar conditions [8,21]. The concentrations of most compounds remained constant before GE (Figure 2), indicating metabolic stability. There were two exceptions: fructose-bisphosphate (FBP) and most of the TCA cycle intermediates. The concentration of FBP increased slightly, but continuously, during the exponential growth phase (Figure 2A). This compound is the product of the reaction catalyzed by phosphofructokinase (PFK), which is the key step of glycolysis. The pool sizes of TCA cycle metabolites decreased during the exponential growth phase. This decrease began as soon as 50 min before GE, while 50% of the initial glucose amount was still present in the medium (Figure 2C). This was the earliest metabolic event observed in these experiments. In contrast, the nucleotide pools remained constant throughout the exponential growth phase (Figure 2D).

Upon GE, drastic changes in the pool size of a large number of metabolites were observed. The concentration of all upper glycolysis metabolites, including FBP, fell dramatically and stabilized only two hours after GE. Similar changes were observed in PPP metabolites (Figure 2B). Both pathways are fed from external glucose, and the drop in their metabolites after GE was expected. Interestingly, the drop in upper glycolytic compounds did not result in a similar drop in metabolites from lower glycolysis. Indeed, the pool sizes of phosphoenolpyruvate (PEP) and 2-/3-phosphoglycerate (2/3PG) increased immediately after GE (Figure 2A). This increase is a consequence of the role of PEP as a phosphate donor for the transport of glucose via the PTS system. Upon GE, the PEP demand for glucose transport suddenly drops, resulting in increased pools sizes of both this compounds and its metabolic precursor, 2/3PG). These compounds returned to their initial concentrations before acetate was completely exhausted. The pool sizes of the TCA cycle metabolites did not significantly change after GE (Figure 2C). Because of the decrease observed before GE, the concentrations of the TCA metabolites during the period of acetate utilization were lower than during the exponential growth phase. The pool sizes of adenosine derivatives decreased moderately after GE, but a marked decrease (to a third of the initial concentrations) in guanosine nucleotides (GMP, GDP and GTP) was observed (Figure 2D). As expected, the concentration of the signaling metabolite cyclic-AMP (cAMP), whose production is known to be triggered by GE, was extremely low before GE, but increased strongly within three min after GE (Figure 2D). Then, the concentration of cAMP remained stable during growth on acetate.

#### 2.1.3. Changes in Gene Expression during the Glucose-Acetate Transition

**Figure 3:**
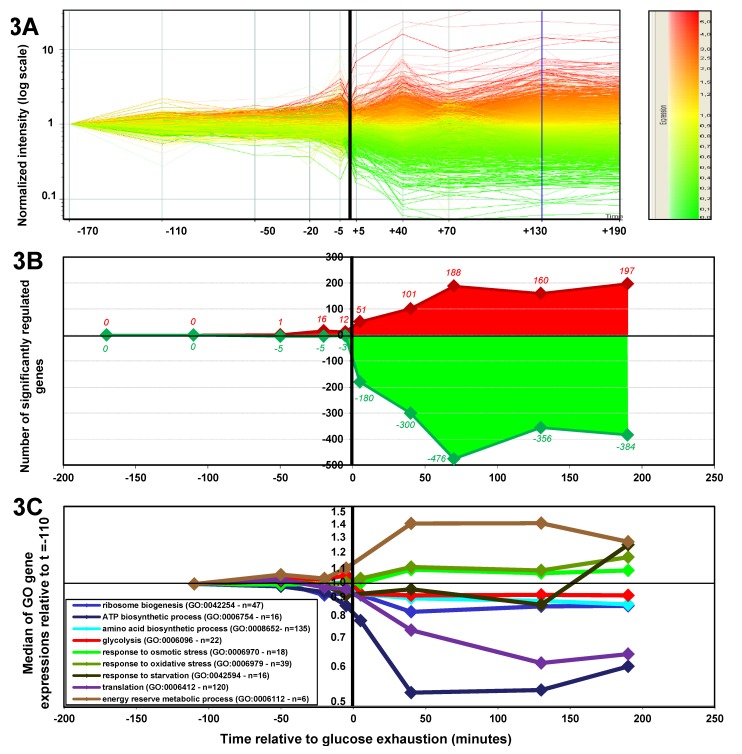
Changes in gene expression during the glucose/acetate transition. (**3A**) The change in *E. coli* genome expression during the transition. Each line represents a change in the expression of a single gene, in comparison with the level of expression measured -170 min before GE. A color scale is used for the expression ratios (toward red when the gene is overexpressed; toward green when it is downregulated). (**3B**) The number of significantly induced or repressed genes per time point (*t*-test < 0.05; red: ratio over 1.5; green: ratio under 0.667). (**3C**) The change in the median of gene expression for selected gene categories. The experiments were performed in three independent biological replicates.

Changes in gene expression during the diauxic transition were monitored by transcriptome analysis. To ensure the consistency of metabolome and transcriptome data, RNA was sampled using the same cultures and sample times as metabolite sampling. For each individual biological replicate, the sample taken in the early exponential phase was used as the reference to evaluate changes in gene expression. The profiles emphasized the stability of gene expression during the exponential growth on glucose. The first variations were detected between 20 and five min before GE (Figure 3A) and increased dramatically in the first five min after GE (Figure 3B). A change in expression in the highest number of genes was observed 70 min later. At that time, roughly 700 genes were up- or down-regulated, *i.e.*, one-sixth of the *E. coli* genome. Interestingly, the expression profile remained stable for three hours, which is also the period of time during which acetate was consumed (Figure 3A). The main functional categories activated or shut down after GE were obtained by calculating the median of the expression of genes belonging to the Gene Ontology terms of interest, as defined in EcoCyc [22]. The results pointed to a massive reshuffling of metabolic and organizational functions after GE (Figure 3C). The main processes during which downregulation was observed upon GE included ribosome biogenesis, ATP biosynthesis, amino acid biosynthesis and glycolysis. Upregulation was observed for stress-response genes (oxidative and osmotic stress genes) and reserve storage genes. For most cellular functions, the reorganization of the transcriptome pattern was completed within 40 min after GE, although some functions, e.g., glycolysis, were readily and stably reset five min after GE. The expression of stationary phase hallmark genes was triggered three hours after GE, which corresponded to when acetate was exhausted (Figure 3C), since no acetate was detected in the medium after that time (data not shown).

#### 2.1.4. Expression of Key Metabolic Genes during the Glucose-Acetate Transition

**Figure 4:**
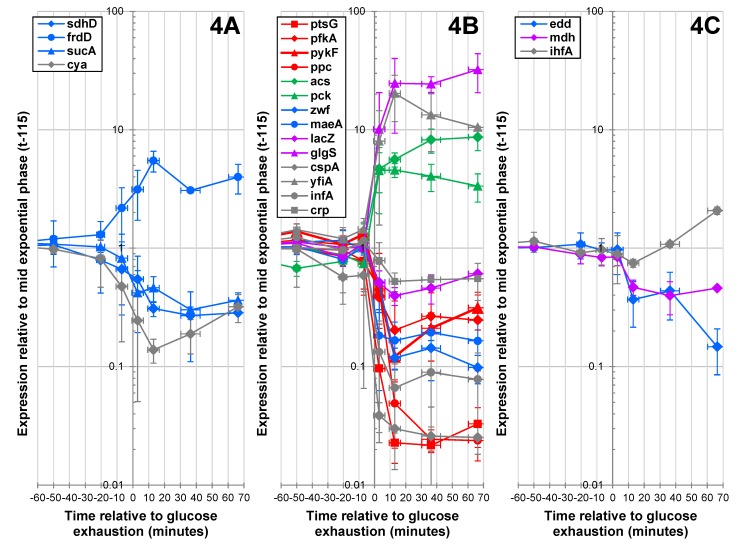
RT-PCR analysis of key gene expressions during the glucose/acetate transition. The plot shows changes in gene expression profiles. Gene expression ratios were normalized according to gene expression levels measured at time −170. (**4A**) Genes up- or down-regulated before GE. (**4B**) Genes up- or down-regulated in the three min following GE. (**4C**) Genes up- or down-regulated more than three min after GE. Glycolytic genes are in red, gluconeogenic genes are in green, blue was used for other central metabolic genes, purple for secondary metabolism genes and gray for non-metabolic genes.

To identify the fine tuning of gene expression around GE, we analyzed the expression of 21 key genes, mainly metabolic genes, during a 2-h time window between 50 min before and 70 min after GE. These investigations were performed by RT-PCR, which is well suited for reliable quantification of small changes in gene expression [23]. The results are shown in Figure 4.

The RT-qPCR data matched the microarray data for the genes investigated using the two methods (Pearson correlation = 0.68 ± 0.07 on average after GE). Genes were classified according to their expression profiles. The first group comprised genes up- or down-regulated before GE. The transcriptomic data highlighted a dozen genes with early regulation. However, changes in expression levels before GE were confirmed by RT-qPCR in only four genes (Figure 4A). The first gene was *cya*, encoding the adenylate cyclase that produces the alarmone, cAMP [24,25]. Interestingly, the three other genes (fumarate reductase, *frdD*, succinate dehydrogenase, *sdhD*, and α-ketoglutarate decarboxylase, *sucA*; Figure 4A) belong to the TCA cycle. The expression of these genes decreased before GE, which was consistent with the early decrease in TCA cycle metabolites observed in the same period. The majority of changes in gene expression observed by RT-qPCR occurred within three min after GE (Figure 4B), in agreement with the microarray data. A dramatic repression of glycolytic and PPP genes, concomitant with the induction of gluconeogenic genes (e.g., *pck*, encoding PEP carboxykinase) was observed. Acetate metabolism genes, such as *acs*, encoding acetate synthase, were also rapidly induced. Interestingly, the expression profiles remained mostly stable over the period of acetate utilization

Only three genes linked to the ED pathway (*i.e.*, *edd*, encoding phosphogluconate dehydratase), to the TCA cycle (*mdh*, encoding malate dehydrogenase) and to the transcriptional regulator, *ihfA* (Figure 4C), regulated later.

#### 2.1.5. Data Integration

**Figure 5:**
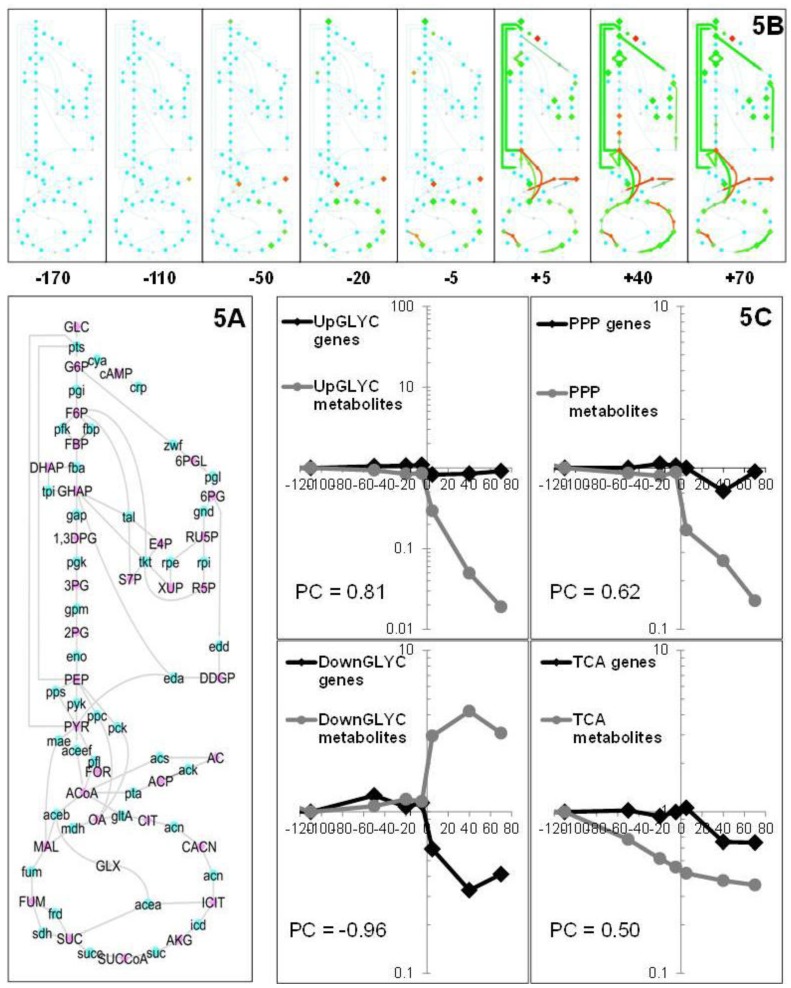
Gene expression and metabolite concentration profiles in the main carbon metabolic pathways. (**5A**) Biochemical network used for the plotting of omics data. This map represents the set of metabolites (blue symbols) and genes (pink symbols) for which the data are plotted. (**5B**) Changes in transcriptome and metabolome data during the glucose-to-acetate transition. Gene expression levels and metabolite ratios are plotted for eight selected time points throughout the transition period and are expressed relative to a reference time (−170 min before glucose exhaustion). The figure was created using Cytoscape software and its MODAM plug as described in Enjalbert *et al.* [26]. Color code: red indicates a positive ratio (higher expression or concentration), green indicates a negative ratio (lower expression or concentration) and yellow indicates unchanged ratios. (**5C**) Changes in the medians of expression/concentration for the genes/metabolites belonging to the same metabolic pathway (the upper or lower glycolysis and pentose phosphate pathway and the TCA cycle). The Pearson correlation score (PC) indicates the similarity of genetic and metabolic variations.

To investigate the correlations between the transcriptome and metabolome data, the two datasets were plotted on a single graph using the visualization tool developed by Enjalbert *et al.* [26]. A time series of maps representing changes in metabolites and transcripts related to central carbon metabolism was generated to analyze the fine tuning of molecular events that occurred during the glucose-acetate transition (Figure 5A). As expected, these representations emphasized the metabolic quasi-steady-state during the exponential growth on glucose, since very few modifications were observed during this phase. The few noticeable changes concerned the metabolome, especially in the TCA cycle. After GE, drastic changes occurred at both the metabolome and transcriptome levels, indicating fast and profound molecular adaptation to the new environmental conditions. To obtain further insight into the correlations between the omics data during the transition period, the median profiles of both metabolite concentrations and gene expressions were calculated and plotted together according to their metabolic pathways. The data (Figure 5B) indicated that the overall decrease in TCA metabolites observed before GE did not correlate with a general repression of TCA cycle genes. These results suggest that the changes in TCA cycle metabolites during the exponential growth phase were not due to gene-level regulation, even though the RT-qPCR data showed that a few TCA genes were downregulated 50 min before GE. The median profiles emphasized the overall decrease in the pool sizes of metabolites from the upper glycolysis and pentose phosphate pathways and the TCA cycle after GE. The median profiles of the upper glycolysis and pentose-phosphate pathways were similar: the overall, marked decrease in metabolite pools did not correlate with a general repression of pathway genes. Interestingly, the profiles obtained for lower glycolysis were strikingly different from those obtained for upper glycolysis, since in the former, there was a general increase in metabolite pools together with a general decrease in gene expression after GE. This suggests genetic control to avoid over-accumulation of metabolites in this part of the pathway. Finally, variations in TCA cycle metabolites were observed before GE, after which the metabolite pools tended to stabilize, whereas TCA genes were repressed.

**Figure 6:**
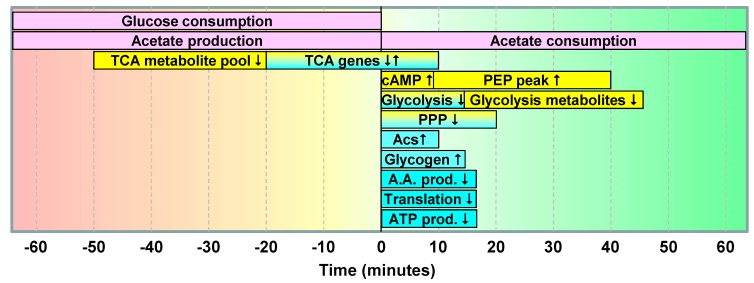
Timing of the glucose/acetate transition.

Pink boxes represent extracellular metabolite variations, Yellow boxes represent metabolite concentrations and blue boxes represent gene expressions.

### 2.2. Discussion

The sequence and timing of the major events that occurred during the glucose-acetate transition, as they appeared in the work reported here, are summarized in Figure 6. The changes in transcriptome and metabolome data can be analyzed in light of the changes in the physiological data.

According to the data reported here, the central metabolism and gene expression pattern were almost stable throughout the initial period of growth on glucose, which is consistent with steady-state metabolism during exponential growth on glucose. By studying the maltose-lactose transition, Mitchell *et al.* [27] recently reported that *E. coli* could anticipate which gene expression was necessary for the use of the second substrate before the first one is completely consumed. This behavior was reported in cells that had been trained for generations to alternate maltose and lactose. Because *E. coli* has been grown for decades on glucose and has certainly evolved to increase its efficiency to grow in such laboratory conditions, it is questionable whether the adaptation to the use of acetate can be anticipated. From the data reported here, in which both metabolism and gene expression were stable, this is likely not the case. The few changes that occurred before glucose exhaustion included a progressive decrease in TCA metabolites and an increase in the FBP/hexose-6-phosphates ratio. Due to the nature of these changes, and to the fact that they were not primarily due to changes in gene expression, it is unlikely that they represent a pre-adaptation to acetate utilization. On the contrary, they are consistent with the onset of the overflow metabolism responsible for acetate production during growth on glucose [6]. Indeed, they indicate a slight imbalance between upper glycolysis and downstream metabolism. Interestingly, the first changes observed, even though they were limited, were metabolic and occurred before changes in gene expression could be detected. This is in agreement with current views placing genetic control after global physiological control mechanisms [28,29].

After complete exhaustion of glucose, the response was extremely fast and massive at both genetic and metabolic levels (Figure 6). Changes in gene expression were observed in up to a sixth of the *E. coli* genome. As expected, biosynthetic and energy conservation machinery was negatively affected by GE, while reserve sugars and stress genes were induced. These changes in gene expression mostly stabilized within the 10 min following GE. The very rapid (within three min) and strong production of cAMP, which is known to regulate the expression of a large number of genes in *E. coli*, is consistent with such rapid genetic adaptation. Such a rapid response could be explained by the onset of adaptive changes in the final min preceding the disappearance of glucose, as hypothesized elsewhere [30,31]. In this context, Ferenci [32] reported that *E. coli* cells were able to co-consume glucose and acetate when the concentration of glucose was set at 0.3 mM or lower during chemostat culture, indicating that functional acetate utilization processes can operate at very low glucose concentrations. In our conditions—*i.e.*, in batch cultures with ODs > 2.5, it would take roughly two min to fully utilize glucose at such a low concentration. As this is below the time resolution of the present investigation, these data do not allow us to conclude on possible co-consumption of the two substrates during the last few min before glucose exhaustion. Nevertheless, our data support the hypothesis of glucose exhaustion being a critical factor in triggering the metabolic transition. The rapidity of the observed molecular changes is also consistent with the capability of *E. coli* to respond to environmental changes within min or even more rapidly [14].

Valgepea *et al.* [33] investigated the growth rate dependency of acetate utilization by glucose-grown cells. They reported that cells growing on glucose at a low growth rate efficiently consumed externally added acetate, but acetate consumption decreased with the growth rate and stopped at μ = 0.48 h^−^^1^ or above. Interestingly, the increase in acetate consumption with decreasing growth rate was shown to be correlated with an increase in cAMP production, the induction of *acs* and a decrease in TCA metabolite concentrations. We made similar observations just after GE, indicating that such changes could be related to the drop in the growth rate as glucose availability decreases. In contrast, the drop in PPP metabolites observed after GE in our experiments was not observed in the experiments performed by Valgepea *et al.* In their experiments, glucose was still available in the feeding medium when acetate was added. The fact that the drop in pentose phosphate pathway metabolites was observed upon GE in our experiments, but not in the experiments by Valgepea *et al.*, suggests that this drop was mainly due to the fact that glucose was not available after GE. Taken together, the above observations suggest that the response observed upon GE in our experiments was triggered by at least two factors: the drop in the growth rate and the disappearance of glucose.

The last molecular changes observed during the glucose-acetate transition were at the level of metabolite pools, which continued to decrease for 40 min after GE. A noticeable exception was PEP and its precursors, which accumulated after glucose exhaustion, likely due to the drop in the demand in PEP for glucose transport and phosphorylation by the PTS system. This means that the adaptation of *E. coli* metabolism to acetate utilization was completed within 40 min after glucose exhaustion, on both genetic and metabolic levels. This is different from the three hours reported by Kao *et al.* [12,13]. In the latter works, cells were grown at 37 °C on glucose until the mid-exponential phase, chilled in cold ethanol, washed at 4 °C and, then, transferred into a pre-warmed medium containing acetate as the sole carbon source. The longer transition period in their work could be partly explained by stress caused by the processes (cell harvest, cold treatment) by which cells were transferred from the first to the second medium. In the present work, we investigated the timing of adaptation without disturbing the culture, meaning that there was no cell harvest and, thus, no stress related to cell collection and cold treatment. It can be reasonably concluded that *E. coli* cells need 40 min to fully adapt to acetate utilization after growth on glucose. Interestingly, this time is similar to that observed for the glucose-lactose transition, in which adaptation to lactose was completed within 50 min after glucose exhaustion [31].

In conclusion, the present work emphasized the rapid massive reshuffling of both gene expression and metabolism just after glucose exhaustion. Detailed information is provided regarding the order, time and extent of the molecular and physiological events that occur during the glucose-acetate transition in *E. coli*, which will be of particular interest in the development of dynamic models of metabolism. The datasets generated in this study are available to the scientific community on the MIAME-compliant EBI database [34]. They are of particular interest in light of recent progress toward comprehensive understanding of metabolism dynamics [8,15].

## 3. Experimental Section

### 3.1. Strain, Media and Growth Conditions

*E. coli* K12 MG1655 was cultured in M9 mineral medium complemented with 2.7 g.L^−1^ (15 mM) of D-glucose [35]. Cultures in shake flasks were performed in a volume of 200 mL or 50 mL. Cultures in bioreactors were inoculated with a washed cell sample obtained from an overnight pre-culture in M9 glucose. Cultures were performed in controlled conditions, using a Multifors bioreactor (Infors, Switzerland). Three independent batches of *E. coli* K12 MG1655 were cultured in 400 mL of M9 mineral medium containing 2.7 g L^−1^ of D-glucose as the sole carbon source. Parameters were locked at pH 7.0, 37 °C, stirring at 800 rpm and air flow at 0.2 L/s to keep the dissolved oxygen tension (DOT) above a minimum threshold of 20%. Throughout the culture, online measurements were performed (DOT, temperature, pH, percentages of CO_2_ and O_2_ in the gas output using BCP gas analyzers - Bluesens, Germany), and pH was set to 7.0 using 1 M solutions of NaOH and HCl. Optical density was measured in triplicate using a spectrophotometer (Genesys6 from Thermo Scientific, Massachusetts). Because the focus of this work was on the glucose-to-acetate switch, RNA and metabolites were sampled before and after glucose exhaustion. The sampling times were defined in preliminary experiments. Samples were taken at 10 minute intervals around glucose exhaustion and at 30 min intervals outside this period. The carbon balance was calculated from the total amounts of all compounds (CO_2_, biomass, acetate, *etc.*) produced and the glucose consumed at the time of glucose exhaustion.

### 3.2. Metabolome Analysis

Exocellular metabolites were identified and quantified by nuclear magnetic resonance (NMR). Broth samples were collected at different times and filtered (Minisart 0.2 µM filter from Sartorius, Göttingen, Germany). The supernatants, corresponding to the culture medium, were mixed with 100 µL of D_2_O containing 2.35 g/L of TSPd4 as the standard (3-(trimethylsilyl)-1-propanesulfonic acid-tetra deuterated). Proton-NMR spectra were recorded on an Avance 500 MHz NMR spectrometer equipped with a 5 mM BBI probe head (Bruker, Rheinstatten, Germany). Spectral processing and metabolite quantification were performed using Topspin 2.1 (Bruker, Rheinstatten, Germany). Extracellular metabolites from three independent biological replicates were analyzed. Three technical samples were collected and analyzed for each biological replicate and at each sampling time.

Intracellular metabolites were sampled by fast filtration as described previously [18]. Briefly, 100 µL of culture were rapidly placed on a filter and washed with ten volumes of diluted cultivation medium containing 1 g L^−1^ of glucose (or acetate after the shift) and frozen in liquid nitrogen. Metabolites were extracted by boiling the filters in 8 mL of H_2_O for 10 min in the presence of 100 µL of a fully ^13^C-labeled cellular extract (used as the internal standard). The solution was filtered, lyophilized and resuspended in 200 µL of ultrapure water. Intracellular metabolites were analyzed as previously described [18,19]. Briefly, analysis was by high performance anion exchange chromatography (Dionex ICS 2000 system, CA, USA) coupled to a triple quadrupole QTrap 4000 (AB Sciex, CA, USA) mass spectrometer. All samples were analyzed in the negative mode by multiple reaction monitoring (MRM). To ensure highly accurate quantification, the isotope dilution mass spectrometry (IDMS) method was used [20]. Metabolome analysis was performed on three independent biological replicates. Three metabolite samples were collected and analyzed for each biological replicate and at each sampling time.

### 3.3. Transcriptome Analysis

Global gene expression levels were determined using DNA-microarrays (3 × 6 K *E. coli* microarray slides, Alberta University, Edmonton, Alberta, Canada). At each sampling time, 2 mg of cell dry weight were harvested by centrifugation (4 °C, 2 min, 5,000 × g) and flash frozen in liquid nitrogen. RNA isolation was performed using the Qiagen RNeasy Midi kit according to the manufacturer’s instructions. The quality and quantity of RNA were checked by capillary electrophoresis (Bioanalyzer from Agilent, Santa Clara, California, USA) following the manufacturer’s instructions. The samples were labeled with Cy5 and a pool of RNA from the first sampling time was labeled with Cy3. The cDNA were co-hybridized on the microarrays, and signals were quantified and assigned to gene names with Genepix software (Molecular Devices Corp.) Normalizations were performed with the Genespring 7 software from Agilent (Lowess and normalization to the first sample to prevent dye swap biases). Transcriptome analysis was performed of three independent biological replicates. 

### 3.4. RT-PCR Analysis

Gene expression of selected genes was also monitored by RT-PCR using the same three independent triplicates. A volume equivalent to 2 mg of cell dry weight was harvested by centrifugation (1 min, 13,000 × g) and flash frozen in liquid nitrogen. Total RNA extractions were performed using a Qiagen RNeasy Midi extraction kit. Samples were treated with DNase to eliminate genomic DNA; the samples were subjected to a reverse transcription using the Super Script II Reverse transcriptase (Life Technology). RT-PCR was performed using the SYBR-Green based detection protocol (Life Technology), with an ICycler real-time PCR detection system (Bio-Rad) and using “MyIQ” software (Bio-Rad). Primers (Table 1) were designed with “primer3” online freeware [36]. Data were normalized *versus* two controls (16S and ihfB genes) and *versus* the mid-exponential phase level of expression.

**Table 1:** RT-PCR primers.

Name	Sequence (5' to 3')	Name	Sequence (5' to 3')
Q-16S-3'	ATCCGGACTACGACGCACTT	Q-lacZ3'	GCATAACCACCACGCTCATCG
Q-16S-5'	ACGACCAGGGCTACACACG	Q-lacZ5'	ACCTACGGGTAACAGTTTCTTTATG
Q-AceA-3'	AACCAGCAGGGTTGGAACG	Q-maeA3'	ATTGCGCGGGAGACTTTCTG
Q-AceA-5'	ACATGGGCGGCAAAGTTTTA	Q-maeA5'	AAGTGAAACGCTGGCGCAGT
Q-aceB-3'	TCAGGCCATAAATCGGCACA	Q-mdh3'	TGGCCCATAGACAGGGTTGC
Q-aceB-5'	GGTGAACGCACCGAAGAAGG	Q-mdh5'	CCGAGCAGGAAGTGGCTGAT
Q-acs-3'	GGATCTTCGGCGTTCATCTC	Q-pck3'	GTGTCTACGCCCGGCAGTTC
Q-acs-5'	GGGAAAATTGACTGGCAGGA	Q-pck5'	GACGCCATCCTCAACGGTTC
Q-crp3'	ACAGGCCCAGTTCGCCAATA	Q-pfkA3'	CACCCATGTAGGAACCGTCA
Q-crp5'	AGGCTCTGTGGCAGTGCTGA	Q-pfkA5'	AATTCCGCGACGAGAACATC
Q-cya3'	CCCGGCGGCACATAAATAAA	Q-ppc3'	CAGGCGAGAACGCAGGTTTT
Q-cya5'	GCCGCGTTTGAAGCATTACC	Q-ppc5'	ATGGTTGAAGCGACCCCTGA
Q-edd3'	ACCAAGTGGCGGCATGAGTT	Q-pps3'	CTGGCTCGTAACGCTCACCA
Q-edd5'	GTTTGCTGGACCGCGATTGT	Q-pps5'	GTGCCGCGTTTTATCCGAAG
Q-frdD3'	CCGCAGGTACGTGGATTTTC	Q-ptsG3'	GGAATGTCGCCGTGGAAAAC
Q-frdD5'	TGGTCGCGTATTCCTGTTCC	Q-ptsG5'	CCGTTTGGTCTGCACCACAT
Q-glgS3'	GCTCTCTTGCCTGCATCATCTG	Q-pykF3'	GCAACCATGATGCCGTCAGA
Q-glgS5'	CGGTCGATATTCTGGCCGTTA	Q-pykF5'	CGGCGAAAACATCCACATCA
Q-Icd-3'	TTCGTCACCGATGTTTGCAC	Q-sdhD3'	ACACACCCCACACCACAACG
Q-Icd-5'	CGCCTGTATGAACCTGAACG	Q-sdhD5'	CGTTAAACCGCTGGCTTTGC
Q-ihfA3'	TACTCGTCTTTGGGCGAAGC	Q-sucA3'	GGTGTCAGGGTCGGAGATCG
Q-ihfA5'	GCGAGGATATTCCCATTACAGC	Q-sucA5'	ACGGGAGTCAAACCGGATCA
Q-ihfB-3'	CAAAGAGAAACTGCCGAAACC	Q-yfiA3'	TGTGGCGTCAGCAACAAACC
Q-ihfB-5'	GCCAAGACGGTTGAAGATGC	Q-yfiA5'	ACCGTCTCGCCAAACTGGAA
Q-infA3'	ACAATGCGGCCTTTGCTCAG	Q-zwf3'	GCCCTTCGATCCCCACTTCT
Q-infA5'	GCACACATCTCCGGTAAAATGC	Q-zwf5'	GGCGCTGCGTTTTGCTAACT

## 4. Conclusions

Knowledge of the molecular mechanisms underlying metabolic adaptations in *E. coli* is still sparse. The regulation, sequence and timing of the events underlying these phases are still poorly understood, as datasets describing the fine timing of metabolic transitions are still rare, even for a classical workhorse like *E. coli*. The main reason for this knowledge gap is the complexity of the regulatory network that controls metabolism, with interwoven genetic and metabolic regulations [8,26,37]; hence the need to combine several omics approaches to address this complexity [38]. Here, we demonstrated the value of multi-omic approaches to further our understanding of metabolic adaptation. Due to the complexity of metabolism and its many layered regulation, this work focused on central carbon metabolism as the first target, but the approach now needs to be extended to the entire metabolic network to get the full picture of adaptation mechanisms.

## Abbreviations

1,3DPG: 1,3-bisphospho-D-glycerate
2/3PG: 2-/3-phosphoglycerate
6PG: 6-phospho D-gluconate
6-PG: 6-phosphogluconate
6PGL: 6-phospho D-glucono-1,5-lactone
a-KG: 2-oxoglutarate
AC: acetate
acea: isocitrate lyase
aceb: malate synthase
aceef: pyruvate dehydrogenase
ack: acetate kinase
acn: aconitate hydratase
ACoA: acetyl-CoA
ACP: acetyl phosphate
acs: acetyl-CoA synthetase
AMP: adenosine-monophosphate
ADP: adenosine-diphosphate
ATP: adenosine-triphosphate
CACN: cis-aconitate
CIT: citrate
crp: CRP transcriptional dual regulator
cya: adenylate cyclase
DHAP: dihydroxyacetone phosphate
E4P: erythrose-4-phosphate
ED: Entner-Doudoroff
eno: enolase
F6P: fructose-6-phosphate
fba: fructose bisphosphate aldolase
FBP: fructose-bisphosphate
Fbp: fructose-1,6-bisphosphatase
FOR: formate
frd: fumarate reductase
fum: fumarase
FUM: fumarate
G6P: glucose-6-phosphate
gap: glyceraldehyde 3-phosphate dehydrogenase
GHAP: D-glyceraldehyde 3-phosphate
GLC: glucose
GLX: glyoxylate
GLYC: glycolysis
gltA: citrate synthase
GMP: guanosine-monophosphate
GDP: guanosine-diphosphate
GTP: guanosine-triphosphate
gnd: 6-phosphogluconate dehydrogenase
gpm: 2,3-bisphosphoglycerate-dependent phosphoglycerate mutase
IC: ionic chromatography
icd: isocitrate dehydrogenase
ICIT: iso-citrate
mae: malate dehydrogenase
MAL: malate
mdh: malate dehydrogenase
MS: mass spectrometry
NAD+: beta-nicotinamide adenine dinucleotide
NMR: nuclear magnetic resonance
OA: oxaloacetate
pck: phosphoenolpyruvate carboxykinase
PEP: phosphoenolpyruvate
Pfk: 6-phosphofructokinase
pfl: pyruvate formate-lyase
pgi: phosphoglucose isomerase
pgk: phosphoglycerate kinase
pgl: 6-phosphogluconolactonase
PP: pentose phosphate
ppc: phosphoenolpyruvate carboxylase
PPP: pentose phosphate pathway
pps: phosphoenolpyruvate synthetase
pta: phosphate acetyltransferase
PTS: phosphotransferase system
pyk: pyruvate kinase
PYR: pyruvate
R5P: D-ribose 5-phosphate
Rib-5P: ribose-5-phosphate
rpe: ribulose-5-phosphate 3-epimerase
rpi: ribose-5-phosphate isomerase
RT-PCR: reverse transcription polymerase chain reaction
RU5P: D-ribulose 5-phosphate
S7P: D-sedoheptulose 7-phosphate
sdh: succinate dehydrogenase
suc: 2-oxoglutarate decarboxylase
SUC: succinate
Succ: succinyl-CoA synthetase
SUCCoA: succinyl-CoA
tal: transaldolase
TCA: Tricarboxylic acid
tkt: transketolase
tpi: triose phosphate isomerase
UMP: uridine-monophosphate
UDP: uridine-diphosphate
UTP: uridine-triphosphate
XUP: D-xylulose 5-phosphate
zwf: glucose 6-phosphate-1-dehydrogenase

## References

[1] Koch A.L. (1971). The adaptive responses of *Escherichia coli* to a feast and famine existence. Adv. Microb. Physiol..

[2] Chang D.E., Smalley D.J., Tucker D.L., Leatham M.P., Norris E., Stevenson S.J., Anderson B., Grissom J.E., Laux D.C., Cohen P.S. (2004). Carbon nutrition of *Escherichia coli* in the mouse intestine. Proc. Natl. Acad. Sci. USA.

[3] Miranda R.L., Conway T., Leatham M.P., Chang D.E., Norris W.E., Allen J.H., Stevenson S.J., Laux D.C., Cohen P.S. (2004). Glycolytic and gluconeogenic growth of *Escherichia coli* O156:H7 EDL933 and *E. coli* K-12 MG1655 in the mouse intestine. Infec. Immun..

[4] Fabich A.J., Jones S.A., Chowdhury F.Z., Cernosek A., Anderson A., Smalley D., McHargue J.W., Hightower G.A., Smith J.T., Autieri S.M. (2008). Comparison of carbon nutrition for pathogenic and commensal *Escherichia coli* strains in the mouse intestine. Infect. Immun..

[5] Papagianni M. (2012). Recent advances in engineering the central carbon metabolism of industrially important bacteria. Microb. Cell. Fact..

[6] Wolfe A.J. (2005). The acetate switch. Microbiol. Mol. Biol. Rev..

[7] Kremling A., Bettenbrock K., Gilles E.D. (2007). Analysis of global control of *Escherichia coli* carbohydrate uptake. BMC Syst. Biol..

[8] Kotte O., Zaugg J.B., Heinemann M. (2010). Bacterial adaptation through distributed sensing of metabolic fluxes. Mol. Syst. Biol..

[9] Peskov K., Mogilevskaya E., Demin O. (2012). Kinetic modelling of central carbon metabolism in *Escherichia coli*. FEBS J..

[10] El-Mansi M., Cozzone A.J., Shiloach J., Eikmanns B.J. (2006). Control of carbon flux through enzymes of central and intermediary metabolism during growth of *Escherichia coli* on acetate. Curr. Opin. Microbiol..

[11] Oh M.K., Rohlin L., Kao K.C., Liao J.C. (2002). Global expression profiling of acetate-grown *Escherichia coli*. J. Biol. Chem..

[12] Kao K.C., Yang Y.L., Boscolo R., Sabatti C., Roychowdhury V., Liao J.C. (2004). Transcriptome-based determination of multiple transcription regulator activities in *Escherichia coli* by using network component analysis. Proc. Natl. Acad. Sci. USA.

[13] Kao K.C., Tran L.M., Liao J.C. (2005). A global regulatory role of gluconeogenic genes in *Escherichia coli* revealed by transcriptome network analysis. J. Biol. Chem..

[14] Sunya S., Delvigne F., Uribelarrea J.L., Molina-Jouve C., Gorret N. (2012). Comparison of the transient responses of *Escherichia coli* to a glucose pulse of various intensities. Appl. Microbiol. Biotechnol..

[15] Schuetz R., Zamboni N., Zampieri M., Heinemann M., Sauer U. (2012). Multidimensional optimality of microbial metabolism. Science.

[16] Sambrook J., Russell D.W. (2011). Molecular Cloning: A Laboratory Manual.

[17] Womack J.E., O’Donovan G.A. (1978). Orotic acid excretion in some wild-type strains of *Escherichia coli* K-12. J. Bacteriol..

[18] Bolten C.J., Kiefer P., Letisse F., Portais J.C., Wittmann C. (2007). Sampling for metabolome analysis of microorganisms. Anal. Chem..

[19] Kiefer P., Nicolas C., Letisse F., Portais J.C. (2007). Determination of carbon labelling distribution of intracellular metabolites from single fragment ions by ion chromatography tandem mass spectrometry. Anal. Biochem..

[20] Wu L., Mashego M.R., van Dam J.C., Proll A.M., Winke J.L. (2005). Quantitative analysis of the microbial metabolome by isotope dilution mass spectrometry using uniformly ^13^C-labeled cell extracts as internal standards. Anal. Biochem..

[21] Chassagnole C., Noisommit-Rizzi N., Schmid J.W., Mauch K., Reuss M. (2002). Dynamic modeling of the central carbon metabolism of *Escherichia coli*. Biotechnol. Bioeng..

[22] Keseler I.M., Collado-Vides J., Gama-Castro S., Ingraham J., Paley S., Paulsen I.T., Peralta-Gil M., Karp P.D. (2005). EcoCyc: A comprehensive database resource for *Escherichia coli*. Nucleic Acids Res..

[23] Wang Y., Barbacioru C., Hyland F., Xiao W., Hunkapiller K.L., Blake J., Chan F., Gonzalez C., Zhang L., Samaha R.R. (2006). Large scale real-time PCR validation on gene expression measurements from two commercial long-oligonucleotide microarrays. BMC Genomics.

[24] Yang J.K., Epstein W. (1983). Purification and characterization of adenylate cyclase from *Escherichia coli* K12. J. Biol. Chem..

[25] Botsford J.L., Harman J.G. (1992). Cyclic AMP in procaryotes. Microbiol. Rev..

[26] Enjalbert B., Jourdan F., Portais J.C. (2011). Intuitive visualization and analysis of multi-omics data and application to *Escherichia coli* carbon metabolism. PLoS One.

[27] Mitchell A., Romano G.H., Groisman B., Yona A., Dekel E., Kupiec M., Dahan O., Pilpel Y. (2009). Adaptive prediction of environmental changes by microorganisms. Nature.

[28] Berthoumieux S., de Jong H., Baptist G., Pinel C., Ranquet C., Ropers D., Geiselmann J. (2013). Shared control of gene expression in bacteria by transcription factors and global physiology of the cell. Mol. Syst. Biol..

[29] Kochanowski K., Sauer U., Chubukov V. (2013). Somewhat in control-the role of transcription in regulating microbial metabolic fluxes. Curr. Opin. Biotechnol..

[30] Ferenci T. (1999). “Growth of bacterial cultures” 50 years on: towards an uncertainty principle instead of constants in bacterial growth kinetics. Res. Microbiol..

[31] Traxler M.F., Chang D.E., Conway T. (2006). Guanosine 3',5'-bispyrophosphate coordinates global gene expression during glucose-lactose diauxie in *Escherichia coli*. Proc. Natl. Acad. Sci. USA.

[32] Ferenci T. (2008). Bacterial physiology, regulation and mutational adaptation in a chemostat environment. Adv. Microb. Physiol..

[33] Valgepea K., Aamberg K., Nahku1 R., Lahtvee P.J., Arike L., Vilu R. (2010). Systems biology approach reveals that overflow metabolism of acetate in *Escherichia coli* is triggered by carbon catabolite repression of acetyl-CoA synthetase. BMC Sys Biol..

[34] Arrayexpress Database. http://www.ebi.ac.uk/arrayexpress/.

[35] Nicolas C., Kiefer P., Letisse F., Krömer J., Massou S., Soucaille P., Wittmann C., Lindley N.D., Portais J.C. (2007). Response of the central metabolism of *Escherichia coli* to modified expression of the gene encoding the glucose-6-phosphate dehydrogenase. FEBS Lett..

[36] Primer3 Input. http://frodo.wi.mit.edu/.

[37] Covert M.W., Palsson B.Ø. (2002). Transcriptional regulation in constraints-based metabolic models of *Escherichia coli*. J. Biol. Chem..

[38] Cho B.K., Charusanti P., Herrgård M.J., Palsson B.Ø. (2007). Microbial regulatory and metabolic networks. Curr. Opin. Biotechnol..

[39] Metatoul home page. http://www.metatoul.fr/.

